# Self-Affirmation Reduces Delay Discounting of the Financially Deprived

**DOI:** 10.3389/fpsyg.2019.01729

**Published:** 2019-07-30

**Authors:** Mehrad Moeini-Jazani, Sumaya Albalooshi, Ingvild Müller Seljeseth

**Affiliations:** ^1^Faculty of Economics and Business, University of Groningen, Groningen, Netherlands; ^2^Department of Leadership and Organization, Kristiania University College, Oslo, Norway; ^3^Department of Leadership and Organizational Behaviour, BI Norwegian Business School, Oslo, Norway

**Keywords:** financial deprivation, poverty, self-threat, self-affirmation, delay discounting, sense of personal control, future-oriented decision-making

## Abstract

Financial deprivation is associated with excessive discounting of delayed rewards. In the present research, we argue that this counterproductive tendency may be driven, at least in part, by the aversive and self-threatening nature of experiencing financial deprivation. Accordingly, we propose that self-affirmation—an intervention known to buffer negative consequences of psychological threats—may reduce delay discounting of the financially deprived. Results of two high-powered, preregistered experiments support this proposition. Specifically, in Study 1 (*n* = 546), we show that among participants with relatively lower income, self-affirmation effectively reduces delay discounting. In Study 2 (*n* = 432), we manipulate the feeling of financial deprivation and demonstrate that self-affirmation reduces delay discounting among those who feel financially deprived. We also examine the underlying process of this effect and find that self-affirmation bolsters a sense of personal control among those who feel financially deprived, which in turn reduces their delay discounting (Study 2). Overall, our findings suggest that the relationship between financial deprivation and delay discounting is malleable and psychological interventions that attenuate self-threats and bolster a sense of personal control can be applied to reduce myopic tendencies of the poor.

## Introduction

Research shows that lack of financial resources increases delay discounting, a tendency to prefer sooner payoffs with smaller values over later payoffs with larger values (Lawrance, 1991; Haushofer and Fehr, 2014; Carvalho et al., 2016; Pepper and Nettle, 2017). This counterproductive tendency manifests itself in myopic behavior of the financially deprived across various decision domains. From finances to education and health, the poor often focus on satisfying their short-term needs and desires instead of securing long-term benefits and payoffs. For instance, low-income people tend to have more debt and less savings (Lea et al., 1993), invest less on education (Blanden and Gregg, 2004), and have unhealthy eating habits and poorer physical health (Ford et al., 1991; Mobley et al., 2006; McLaren, 2007). Critically, increased delay discounting systematically impedes the poor’s ability to act in line with their long-term interests, which over time can make the poor’s disadvantaged situation an inescapable constituent of their reality (Vohs, 2013; Farah and Hook, 2017).

Although the relationship between financial deprivation and delay discounting is well-established in the literature, surprisingly little is known about the *psychological interventions* that might mitigate this detrimental tendency among the financially deprived. Identifying such interventions is crucial from both theoretical and practical perspectives. From a theoretical perspective, such discoveries will help elucidate the *psychological* processes through which financial deprivation drives myopic decisions and shed light on ways to counter them. Furthermore, such discoveries will underscore the malleable nature of the relationship between financial deprivation and delay discounting and thus go beyond the deterministic perspectives that relate excessive discounting tendency of the poor mainly to immutable variables such as genetic and personality dispositions (Rowe and Rodgers, 1997; Webley and Nyhus, 2001), or neighborhood structure (Harding, 2003; Wen et al., 2003). Identifying psychological interventions to reduce delay discounting of the financially deprived is also relevant from a public policy perspective, primarily because such interventions, once empirically verified, are relatively easy and inexpensive to implement in poverty alleviation programs. Importantly, such interventions may enable the financially deprived to psychologically distance themselves from their dire circumstances and escape poverty over time.

The present work aims to contribute to the literature by proposing and demonstrating that self-affirmation—cultivating a sense of self as worthy, adequate, and efficacious by affirming one’s core personal values (Steele, 1988; Cohen and Sherman, 2014)—is one such intervention that reduces delay discounting of the financially deprived. In the following sections, we begin our theorizing by reviewing the research suggesting that the self-threatening and aversive nature of financial deprivation may be responsible, at least partly, in how it causes counterproductive and myopic decision tendencies. Bridging between those findings and the literature on self-affirmation theory, we then make a case for why and how self-affirmation may mitigate delay discounting of the financially deprived. Subsequently, we report the results two high-powered, pre-registered experiments testing our propositions. Finally, we discuss the contributions of our findings and their implications for future research.

### Delay Discounting and the Self-Threatening Nature of Financial Deprivation

Money is a fundamental resource that provides access to rewards, both physical (e.g., food) and social (e.g., prestige), and facilitates achieving goals in everyday life (Lea and Webley, 2006; Fritsche and Jugert, 2017). Lacking financial resources, therefore, constitutes a direct threat to people’s innate need to view themselves as capable of overcoming challenges and achieving desired outcomes in daily life (Hughes and Demo, 1989; Boardman and Robert, 2000; Fritsche and Jugert, 2017; Cannon et al., 2018). This conception corroborates with research showing that the financially deprived experience more stress and uncertainty, feel less power to influence their environment, and perceive more difficulty to accomplish daily tasks (Lachman and Weaver, 1998; Cohen S. et al., 2006; Haushofer and Fehr, 2014; Piccolo et al., 2014; Robinson and Piff, 2017).

When people’s prospect of achieving desired outcomes in the future is threatened, maximizing outcomes in the present takes priority, leading to systematic *future neglect* across decision domains. This notion implies that the myopic and counterproductive tendencies displayed by the poor may be driven, at least in part, by the self-threatening nature of financial deprivation. Evidence supporting this theorizing comes from experimental findings in which participants, independent of their actual income and socioeconomic characteristics, were randomly assigned to treatment conditions where the *feeling* of financial deprivation was manipulated. People who *felt* they were financially deprived were more likely to prefer smaller, sooner over larger, later monetary rewards (Callan et al., 2011), save less and borrow more (Shah et al., 2012), and consume more calorific food (Briers and Laporte, 2013).

To the extent that delay discounting of the poor is driven by the self-threatening nature of lacking financial resources, a different scenario may occur if the financially deprived have the opportunity to restore their sense of self-worth—a global and positive perception of the self as adequate, capable, and efficacious. Specifically, such an opportunity may enable the financially deprived to feel capable of achieving desired outcomes despite their dire state, and thus shift their attention from the present and now toward the future. Following this reasoning and drawing on the psychology of self-defense (Cohen and Sherman, 2014), we propose that self-affirmation, an intervention known to buffer psychological threats, may reduce delay discounting of the financially deprived.

### Self-Affirmation: A Remedy for Delay Discounting of the Financially Deprived

Self-affirmation is one of the most frequently studied interventions, known to neutralize the adverse effects of psychological threats. Self-affirmation theory hinges on the premise that the self-system is flexible to the extent that when the self is threatened in one domain, affirming the self in a different domain restores a sense of self-worth and adequacy, that can be harnessed to buffer the detrimental effects of psychological threats (Steele, 1988; Sherman and Cohen, 2006; Cohen and Sherman, 2014). As Steele (1988) states, self-affirmation is a strategy to bolster and appraise the self as “competent, good, coherent, unitary, stable, capable of free choice, capable of controlling important outcomes” (p. 262).

Relating to the present research, a wealth of research has shown that self-affirmation fosters future-oriented tendencies and behaviors among people of low socioeconomic status (SES). For example, self-affirmation has been found to motivate minority students to take more challenging courses, earn better grades, and achieve higher levels of college enrollment, despite their economic disadvantages and existing negative stereotypes about their intellectual abilities (Sherman et al., 2013; Harackiewicz et al., 2014; Goyer et al., 2017). Moreover, research has found that self-affirmation can reduce defensive reactions to health-risk information among low-income smokers with high health risk and enhance their tendency to adopt healthier habits (Armitage et al., 2008). Furthermore, self-affirmation among the poor has been found to improve executive control—a set of fundamental cognitive processes underlying self-regulation, planning, and goal-directed behavior (Hall et al., 2014).

The reviewed findings suggest that self-affirmation can be effective in shifting the attention of the financially deprived from satisfying short-term needs toward securing more long-term benefits, a capacity which is crucial for decisions concerning intertemporal tradeoffs. This proposition is also consistent with recent neuroscientific evidence revealing that the brain regions involved during self-affirmation significantly overlap with the brain areas associated with thinking about the future and prospection (Cascio et al., 2015). Accordingly, we propose:

*Hypothesis 1 (H1)*: Self-affirmation moderates the relationship between financial deprivation and delay discounting, such that among the financially deprived (vs. financially non-deprived) self-affirmation significantly reduces delay discounting.

### How Does Self-Affirmation Foster Future-Oriented Tendencies?

To examine the underlying process of our first hypothesis, we adhere to the core of how self-affirmation augments *motivation* to buffer the detrimental consequences of self-threats. Specifically, whereas lacking monetary resources is invariably self-threatening, self-affirmation bolsters a self-view that is resourceful and capable of overcoming challenges by reminding people of psychosocial resources residing in them (Steele, 1988). This reinstated positive self-view subsequently generates a more adaptive response to threats (Cohen and Sherman, 2014). Notably, the belief that one can influence their environment and achieve desired outcomes despite challenges is the essence of the feeling of personal control (Skinner, 1996; Lachman and Weaver, 1998), which is a crucial determinant of self-regulation and future-oriented intentions and behavior (Schwarzer, 1992). Particularly, sense of control has been found to facilitate goal achievement and performance (Bandura and Wood, 1989; Wood and Bandura, 1989; Karniol and Ross, 1996) and is a positive predictor of one’s savings (Perry and Morris, 2005; Cobb-Clark et al., 2016), health and well-being (Lachman and Weaver, 1998). The positive relationship between sense of control and self-regulatory abilities is also consistent with research showing that increased sense of control is associated with greater optimism (Marshall and Lang, 1990), cognitive control abilities (Schmid et al., 2015; Albalooshi et al., 2019), and abstract construal (Smith and Trope, 2006), which are imperative for goal pursuit and future-oriented intentions and behavior.

Relating to the present work, a greater sense of control, resulting from self-affirmation, may enable the financially deprived to allocate mental resources to a more distant future, which in turn, reduces their tendency to discount larger-later payoffs. This prediction dovetails with the several findings in the past, highlighting the role of sense of control as a mechanism through which self-affirmation extends its reparative effects. For example, research shows that self-affirmation increases perceived personal control among people at high health-risk, which in turn fosters their tendency to adopt healthier habits (Reed and Aspinwall, 1998; Harris et al., 2007; Armitage et al., 2008; Epton et al., 2015). Similarly, in organizations undergoing downsizing where employees often experience high levels of job insecurity, self-affirmation has been found to reduce stress by bolstering employees’ perceived agency in coping with workplace challenges (Morgan and Harris, 2015). Most relevant to the current theorizing, recent research shows that self-affirmation among people who feel powerless boosts a sense of control, which in turn improves their cognitive control abilities (Albalooshi et al., 2019). Accordingly, we propose:

*Hypothesis 2 (H2)*: A perceived sense of personal control mediates the interaction between the financial deprivation and self-affirmation on delay discounting.

Finally, in delineating the underlying process, we also explore the potential role of affect. Although evidence for an affect-based explanation of self-affirmation effects is relatively limited (for a detailed discussion see McQueen and Klein, 2006; Cohen and Sherman, 2014), earlier conceptualizations have highlighted affect regulation as a core mechanism of self-affirmation (Tesser, 2000). Concerning our research, this theoretical perspective implies that self-affirmation may increase positive (or decrease negative) affect among the financially deprived. Notably, positive affect has been found to reduce delay discounting, the primary outcome variable in our research (Pyone and Isen, 2011). Accordingly, in the present research, we measure and examine the role of affect, as an alternative motivational account to our second hypothesis, on how self-affirmation may reduce delay discounting of the financially deprived.

### Overview of the Studies

In two experiments, we test whether self-affirmation reduces delay discounting of the financially deprived (H1; Studies 1 and 2) and examine the role of sense of personal control as the underlying motivational process of this effect (H2; Study 2). Before running the experiments, we registered our hypotheses, the methods, and analyses plans on the Open Science Framework.^1^ In Study 1, utilizing income to index financial deprivation, we demonstrate that among participants with relatively lower income, self-affirmation effectively reduces delay discounting. We replicate these findings in Study 2 by manipulating feeling of financial deprivation. Moreover, in Study 2, we also show that a boost in the sense of personal control explains the mitigating effect of self-affirmation on delay discounting of the financially deprived.

As specified in our preregistered plans, the sample size for each study was determined *a priori* using G^*^Power (v 3.1; Faul et al., 2009) to have a power of 0.80 and an α-error probability of 0.05 to detect the hypothesized effect. In Study 1, power analysis for a linear regression yielded a minimum sample of 387 to detect a small-sized interaction effect (*R*^2^_partial_ = 0.02) between income (measured) and self-affirmation (manipulated) on delay discounting. Likewise, in Study 2, power analysis for an ANOVA yielded a minimum sample of 387 to detect a small-sized two-way interaction (η^2^_partial_ = 0.02) between financial deprivation (manipulated) and self-affirmation (manipulated) on delay discounting. Consequently, for each study, we aimed to sample at least the minimum number of participants determined by the power analysis, with more participants being included if our allotted budget would allow. Sample sizes for both studies exceeded these minima. For each experiment, data were collected in a single attempt and analyzed only after all measures had been collected.

Participants in our studies were American residents recruited via the online crowdsourcing platform, Amazon Mechanical Turk (MTurk). To ensure data quality, we used TurkPrime as a medium to recruit MTurk participants (Litman et al., 2017). MTurk participants are generally diverse in race, gender, education, and income levels, making MTurk a suitable platform for conducting psychological studies (Buhrmester et al., 2011; Berinsky et al., 2012; Crump et al., 2013), and studies related to SES and decision-making (Tully et al., 2015). Finally, for the present research, we have disclosed all manipulations, measures, and exclusions used in our studies. Particularly, where relevant, we refer the reader to the extensive Supplementary Material accompanying this article, which provides details of all materials and measures, as well as, additional analyses of our data.

## Study 1

Income is a common measure of one’s financial status. Importantly, people with relatively low income feel more financially constrained, are frequently concerned about cost and money in daily life (Shah et al., 2018), and have a higher tendency to discount larger-later payoffs (Haushofer and Fehr, 2014). Therefore, to provide a stringent test of our H1, and in order to increase the external validity of our research, we use participants’ income to operationalize financial deprivation in Study 1. Specifically, we test whether self-affirmation is effective in reducing delay discounting of people with the relatively low (vs. high) income.

### Method

#### Participants

Five hundred eighty participants took part in a 2 (affirmation: self-affirmation vs. no-affirmation; between-subjects) × income (measured) experiment.

#### Procedure

We manipulated self-affirmation using the standard value-affirmation procedure (Cohen G.L. et al., 2006; McQueen and Klein, 2006; Sherman et al., 2009). Accordingly, participants were first randomly assigned to either a *self-affirmation* or a *no-affirmation* condition. In both conditions, participants first ranked a list of values (e.g., relations with friends/family, sense of humor) based on their personal importance. Subsequently, in the *self-affirmation* condition, participants wrote about why their top-ranked value was personally important to them. Conversely, in the *no-affirmation* condition, participants wrote about why their least important value might be important to an average university student. Therefore, by contemplating on a belief they did not firmly hold, these participants did not have an opportunity to self-affirm (McQueen and Klein, 2006).

Following the value affirmation task, participants completed the Positive and Negative Affective Schedule (PANAS; Watson et al., 1988) using 5-point scales (1 = not at all, 5 = extremely). The PANAS consists of 20 items capturing both positive (10 items) and negative (10 items) affective states. For each participant, responses to positive (α = 0.91) and negative (α = 0.91) affective states were averaged separately to form positive and negative affect indexes, respectively.

Next, participants completed a delay discounting task. Specifically, using three intertemporal questions (e.g., $65 now = $ – in 3 months), participants indicated the amount of money (in U.S. dollars) they would require in 3, 9, and 18 months in the future, to make them indifferent to receiving $65 now (for a comparable procedure see Kim and Zauberman, 2012). The discounting questions were presented individually and in random order. Following Myerson et al. (2001), we calculated participants’ delay discounting using the area under the discounting curve (AUC) method. The AUC provides a single statistic that does not depend on theoretical assumptions regarding the form of the discounting function and can be easily used to compare experimental groups (Myerson et al., 2001). The AUC can vary between 0 (steepest possible discounting) and 1 (no discounting), where a bigger number indicates a higher preference for larger-later payoffs (i.e., decreased delay discounting).

In the last part of the survey, we measured demographic variables. In particular, we measured participants’ annual income using 20 income brackets with $10,000 increments, ranging from 1 (under $10,000) to 20 (190,000 or more). Following recommendations in analyzing income data (Hout, 2004), we assigned the midpoint of the chosen category as the corresponding individual’s income amount. For example, we assigned the income value of $45,000 to participants who chose the fifth category ($40,000–$49,999). Using linear extrapolation^2^, we derived and considered $195,000 as the income value for individuals who chose the highest income category ($190,000 or more). For the easier interpretation of the results, we divided income values by 10,000.

In addition to income, we measured participants’ demographic characteristics that may covary with income. These included: *age*, *gender*, *ethnicity, level of education, employment status*, and *household size*. As our preregistered plan highlights, we measured these demographic variables to test the robustness of our main findings.^3^ Finally, participants completed an attention check question (Oppenheimer et al., 2009), were debriefed, and paid (see Supplementary Material for details of instructions, measures, and procedures used in this study).

### Results

#### Data Inspection

Based on our preregistered screening criteria, we excluded 34 participants before data analysis who either did not complete the survey (*n* = 8), did not follow instructions and wrote irrelevant texts (*n* = 17), or failed the attention check question (*n* = 9). Therefore, the final analysis was conducted on 546 participants (M_age_ = 38.33, SD = 12.62; 310 females), providing sufficient statistical power to test the hypothesized interaction effect between self-affirmation and income on delay discounting.

#### Income, Self-Affirmation, and Delay Discounting

**FIGURE 1:**
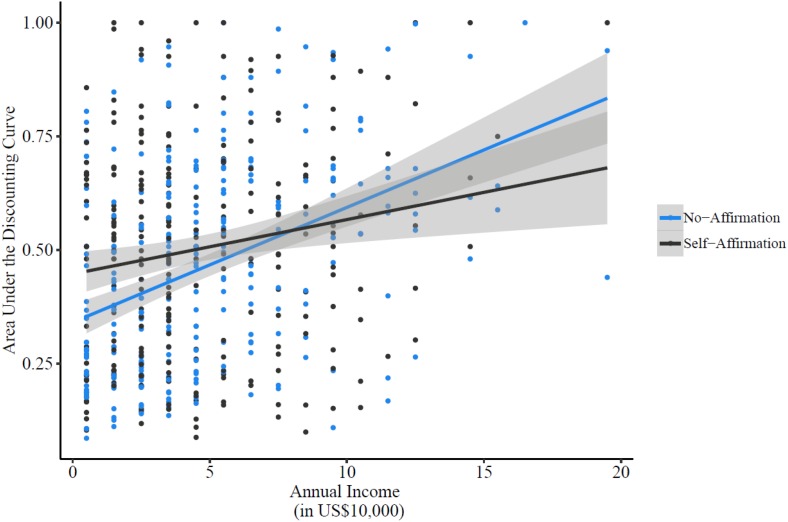
The area under the discounting curve (AUC) as a function of income and affirmation conditions in Study 1. A greater AUC indicates a higher preference for larger-later payoffs (i.e., reduced delay discounting). Error bands denote 95% confidence intervals of the regression estimates.

We regressed participants’ AUC on affirmation (dummy coded, 0 = no-affirmation, 1 = self-affirmation), income^4^ (mean centered), and their interaction term (see Figure 1). Results revealed a significant main effect of income, *b* = 0.025, SE*_*b*_* = 0.004, *t*(542) = 7.10, *p* < 0.001, 95% CI [0.018, 0.032], a main effect of affirmation, *b* = 0.043, SE*_*b*_* = 0.019, *t*(542) = 2.31, *p* = 0.021, 95% CI [0.006, 0.080], and the critical interaction between income and affirmation, *b* = −0.013, SE*_*b*_* = 0.005, *t*(542) = −2.52, *p* = 0.012, 95% CI [−0.024, −0.003].

Following Aiken and West (1991), using a series of regressions, we probed this interaction by examining the effects of affirmation on AUC at one standard deviation above (high) and below (low) the grand mean of income. As expected, among participants with relatively lower income in our sample (M_Income_ – 1 SD), affirming core personal values, relative to no-affirmation condition, significantly increased AUC (i.e., reduced delay discounting), *b* = 0.091, SE*_*b*_* = 0.026, *t*(542) = 3.42, *p* < 0.001, 95% CI [0.038, 0.143]. Conversely, among participants with relatively higher income in our sample (M_Income_ + 1 SD), affirming core personal values, relative to no-affirmation condition, did not further increase AUC, *b* = −0.004, SE*_*b*_* = 0.026, *t*(542) = −0.16, *p* = 0.87, 95% CI [−0.056, 0.048].

Consistent with our H1, these findings demonstrate that self-affirmation is particularly effective in reducing delay discounting among relatively low-income people. To pinpoint the range of income values for which the mitigating effect of self-affirmation (vs. no-affirmation) on delay discounting was significant (α = 0.05 criterion) in our sample, we further probed the interaction between income and affirmation on AUC using the Johnson–Neyman technique (Johnson and Neyman, 1936; Hayes, 2017), while correcting for the potential false positive discovery rate, following the recommendations of Esarey and Sumner (2018). Results of this analysis revealed an income value of $43,300 as the transition point. More specifically, relative to no-affirmation, self-affirmation significantly reduced delay discounting of people whose annual income was smaller than $43,300. However, self-affirmation (vs. no-affirmation) did not significantly reduce delay discounting of people whose annual income was higher than $43,300 in our sample.

#### Robustness Checks

Using a series of regression analyses (see Table 1), we tested whether the focal interaction between income and self-affirmation (Model 1) remained significant after controlling for participants demographic characteristics that can covary with income (Models 2 and 3). Notably, we controlled for participants’ age and gender in Model 2, and for ethnicity, level of education, employment status, and household size in Model 3. As the results of these analyses in Table 1 show, the critical interaction between income and self-affirmation on AUC remained significant even after controlling for participants’ socioeconomic and demographic characteristics. These results, therefore, corroborate the robustness of our main findings.

**TABLE 1:** Robustness tests for Study 1.

	**Model 1**			**Model 2**			**Model 3**		
**Variable**	***b***	**SE*_*b*_***	***t***	***b***	**SE*_*b*_***	***t***	***b***	**SE*_*b*_***	***t***
Intercept	0.462	0.013	35.28^∗∗∗^	0.503	0.016	30.61^∗∗∗^	0.464	0.031	14.83^∗∗∗^
Income	0.025	0.004	7.06^∗∗∗^	0.023	0.004	6.45^∗∗∗^	0.020	0.004	5.28^∗∗∗^
Affirmation	0.043	0.019	2.27^*^	0.045	0.019	2.41^*^	0.045	0.018	2.46^*^
Income × Affirmation	–0.013	0.005	−2.50^*^	–0.012	0.005	−2.21^*^	–0.012	0.005	−2.33^*^
Age				0.002	0.001	2.10^*^	0.001	0.001	1.84^†^
Gender				–0.075	0.019	–3.98^∗∗∗^	–0.076	0.019	–4.07^∗∗∗^
Ethnicity							0.034	0.022	1.53
Education level							0.032	0.009	3.41^∗∗∗^
Employment status							0.017	0.024	0.73
Household size							0.003	0.007	0.50
Adjusted *R*^2^	0.101			0.129			0.145		
*R*^2^ change				0.030^∗∗∗^			0.023^∗∗^		

The area under the discounting curve (AUC) serves as the dependent variable. Regression coefficients are unstandardized. For model 1, *n* = 546. For models 2 and 3, *n* = 544 because we excluded participants who had not classified their gender as male or female (*n* = 2). Income was divided by 10,000 and then centered at its grand mean. Affirmation was coded 1 = self-affirmation, and 0 = no-affirmation. Gender was coded 1 = female and 0 = male. Ethnicity was coded 1 = European–American, and 0 = others. Employment status was coded 1 = full-time, part-time, or self-employed, and 0 = unemployed. Age, education level, and household size were centered at their grand means. *t* statistics are rounded to two digits after the decimal point. ^†^*p* < 0.10, ^*^p < 0.05, ^∗∗^*p* < 0.01, ^∗∗∗^*p* < 0.001.

#### Testing the Role of Affect

We examined whether income and affirmation influenced participants’ affective state. In doing so, we subjected participants’ positive and negative affect scores to two separate regressions with income (centered), self-affirmation (0 = no-affirmation, 1 = self-affirmation) and their interaction term as independent variables. As the results of these analyses in Table 2 demonstrate, the interaction between income and affirmation was not significant in predicting the positive or the negative affect. Furthermore, when controlling for participants’ positive and negative affect in our main model with AUC as the dependent variable, they did not significantly predict AUC while the interaction between income and self-affirmation remained significant (see Table 2). These results suggest that affect regulation is unlikely to explain how self-affirmation reduces delay discounting of people with low income.

**TABLE 2:** Testing the role of affect in Study 1.

**Outcome**	**Positive affect**			**Negative affect**			**AUC**		
**Predictors**	***b***	**SE*_*b*_***	***t***	***b***	**SE*_*b*_***	***t***	***b***	**SE*_*b*_***	***t***
Intercept	2.966	0.052	56.51^∗∗∗^	1.369	0.032	42.36^∗∗∗^	0.460	0.013	35.12^∗∗∗^
Income	0.020	0.014	1.37	–0.024	0.009	–2.68^∗∗^	0.025	0.004	7.01^∗∗∗^
Affirmation	0.218	0.075	2.90^∗∗^	–0.081	0.046	−1.75^†^	0.044	0.019	2.35^*^
Income × Affirmation	–0.011	0.021	–0.53	0.017	0.013	1.28	–0.013	0.005	−2.50^*^
Positive affect							–0.011	0.011	–0.98
Negative affect							–0.013	0.017	–0.77
Adjusted *R*^2^	0.014			0.014			0.101		
*R*^2^ change due to affect covariates							0.002^n.s.^		

Regression coefficients are unstandardized. In all models, *n* = 546. Income was divided by 10,000 and then centered at its grand mean. Affirmation was coded 1 = self-affirmation, and 0 = no-affirmation. As covariates, positive and negative affect were centered at their grand mean. *t* statistics are rounded to two digits after the decimal point. n.s. denotes not significant. ^†^*p* < 0.10, ^*^p < 0.05, ^∗∗^*p* < 0.01, ^∗∗∗^*p* < 0.001.

### Discussion

Results of Study 1 are consistent with our H1. Specifically, we found that, an opportunity to restore self-worth by affirming core personal values, effectively reduced delay discounting of low-income people. In line with our theorizing, these results indicate that the detrimental effect of lacking financial resources on delay discounting is driven, at least partly, by the psychological threat that financial deprivation poses to one’s self-worth.

Notably, the mitigating effect of self-affirmation on delay discounting of low-income people was robust and remained significant even after controlling for demographic variables that often covary with one’s financial status. Nevertheless, despite its promising results, Study 1 has a few shortcomings that need to be addressed. First, although using income as a measure of financial deprivation increases the external validity of our findings, we did not manipulate the feeling of financial deprivation directly. Second, whereas we ruled out the role of affect as the underlying process of our findings, we did not test for the underlying process we proposed in H2: *increase in the sense of control*. In Study 2, we address these limitations.

## Study 2

The aim of Study 2 is to conceptually replicate our main findings in Study 1, by manipulating feeling of financial deprivation, and to directly test for the sense of control as the underlying mechanism of our effect.

### Method

#### Participants

Four hundred and fifty American participants took part in a 2 (financial status: deprived vs. non-deprived) × 2 (affirmation: self-affirmation vs. no-affirmation) between-subjects experiment.

#### Procedure

We adopted a well-established procedure used in the past research to manipulate feeling of financial deprivation (Nelson and Morrison, 2005; Haisley et al., 2008; Callan et al., 2011; Briers and Laporte, 2013). In brief, our procedure consisted of a response scale followed by false feedback on participants’ financial status, and a subsequent writing task. Participants first specified their gender, age, and household size. They were then randomly assigned to either a *financially deprived* or a *financially non-deprived* experimental condition where they had to indicate their “monthly income” using a response scale. In the *financially deprived* condition, participants viewed a sliding scale ranging from $0 to $50,000 (and above), with $5,000 increments. In the *financially non-deprived* condition, participants viewed a sliding scale ranging from $0 to $2,000 (and above), with $200 increments. When participants respond toward the top or bottom of a scale, they tend to make corresponding inferences about their circumstances (Schwarz, 1999). Therefore, people responding to the $50,000 scale should experience a relative feeling of financial deprivation. Conversely, people responding to the $2,000 scale should experience a relative feeling of financial adequacy and sufficiency.

After entering their information, participants were informed that an algorithm would calculate their relative financial status, by comparing their income with people matching their profile, from a large, representative national sample of income data. In reality, no comparison took place, and all participants received bogus feedback on their relative financial status. Particularly, participants in the *financially deprived* condition—those who responded to the $50,000 scale—were told that they lacked financial resources, relative to others, and were asked to think and write about how it feels to live a financially constrained life. In contrast, participants in the *financially non-deprived* condition—those who responded to the $2,000 scale—were told that they had an adequate and sufficient amount of financial resources, relative to others. They were then asked to think and write about how it feels to live a financially adequate life (see Supplementary Material for a detailed description of this manipulation and its pretest^5^). After the financial deprivation manipulation, participants were randomly assigned to one of the two affirmation conditions (self-affirmation vs. no-affirmation) using the same procedure outlined in Study 1.

Immediately after the self-affirmation manipulation, participants completed the 12-item *sense of personal control* scale (Lachman and Weaver, 1998), using 7-point scales (1 = strongly disagree, 7 = strongly agree). This scale captures the extent to which people perceive themselves as agentic and capable of influencing their environment and carrying out goals despite challenges (e.g., “what happens to me in the future mostly depends on me”). After reverse codings and subsequently checking the reliability of the scale (α = 0.93), we averaged across all items to derive participants’ scores on the sense of control. In our analysis, a higher score indicates a higher sense of personal control. Additionally, as in Study 1, participants’ positive (α = 0.92) and negative (α = 0.93) affective states were measured using the PANAS (Watson et al., 1988).

Next, we administered the same procedure outlined in Study 1 to measure and calculate participants’ delay discounting. As in Study 1, the AUC served as our dependent variable. Subsequently, we measured participants’ income and demographic characteristics (e.g., ethnicity, level of education, and employment status) using the same scales in Study 1. As highlighted in our preregistered plan, we measured these variables to test the robustness of our main findings (see Supplementary Material for details of these analyses). Finally, participants completed an attention check question (Oppenheimer et al., 2009), were debriefed, and paid (see Supplementary Material for details of instructions, measures, and procedures used in this study).

### Results

#### Data Inspection

Based on the screening criteria specified in our preregistered plan, we excluded 18 participants before data analysis as they either did not complete the survey (*n* = 7) or failed the attention check question (*n* = 11). The final analysis was therefore conducted on 432 participants (M_age_ = 36.55, SD = 11.19; 253 females), providing sufficient statistical power to test the hypothesized interaction effect between financial deprivation and self-affirmation.

#### Financial Deprivation, Self-Affirmation, and Delay Discounting

**FIGURE 2:**
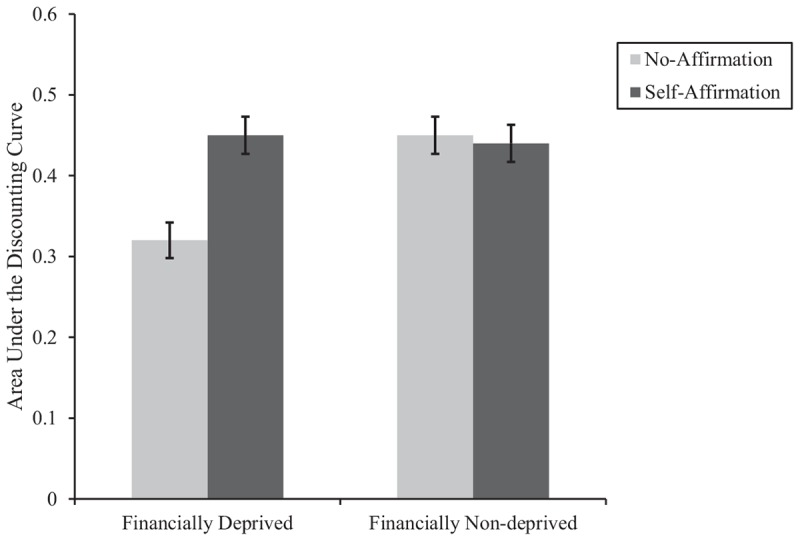
The area under the discounting curve (AUC) for each experimental condition in Study 2. A larger AUC indicates a higher preference for larger-later payoffs (i.e., reduced delay discounting). Error bars denote ±1 SEM.

We subjected participants’ AUC to a 2 (financial status: deprived vs. non-deprived) × 2 (affirmation: self-affirmation vs. no-affirmation) between-subjects ANOVA. Results revealed a main effect of financial status, *F*(1,428) = 7.53, *p* = 0.006, η^2^*_*p*_* = 0.02, a main effect of affirmation, *F*(1,428) = 6.77, *p* = 0.01, η^2^*_*p*_* = 0.02, and the critical two-way interaction between financial status and affirmation, *F*(1,428) = 8.55, *p* = 0.004, η^2^*_*p*_* = 0.02 (see Figure 2).

Analysis of simple effects revealed that, consistent with our first hypothesis (H1), in the deprived condition, participants who affirmed their core personal values (M = 0.45, SD = 0.26) showed less delay discounting (i.e., larger AUC) than did those in the no-affirmation condition, M = 0.32, SD = 0.20; *F*(1,428) = 15.77, *p* < 0.001, *d* = 0.54, 95% CI _Mean–Difference_ [0.06, 0.19]. However, in the non-deprived condition, there was no significant difference in delay discounting between those who affirmed (M = 0.44, SD = 0.24) and those who did not, M = 0.45, SD = 0.23; *F* < 1, *p* = 0.82, *d* = 0.03, 95% CI _Mean–Difference_ [−0.07, 0.06].

Looked at differently, in the no-affirmation condition, participants who felt financially deprived (M = 0.32, SD = 0.20) showed increased delay discounting (i.e., smaller AUC) compared to those who did not feel financially deprived, M = 0.45, SD = 0.23; *F*(1,428) = 16.26, *p* < 0.001, *d* = 0.59, 95% CI _Mean–Difference_ [−0.19, −0.07], replicating past findings that feeling financially deprived increases delay discounting. Interestingly, however, among those who affirmed their core personal values, there was no significant difference in delay discounting whether they felt financially deprived (M = 0.45, SD = 0.26) or not, M = 0.44, SD = 0.24; *F* < 1, *p* = 0.90, *d* = 0.02, 95% CI _Mean–Difference_ [−0.06, 0.07], suggesting that self-affirmation eliminated the existing gap in delay discounting between those who felt financially deprived and those who did not.^6^

Together, these findings corroborate the central proposition of this research. That is, self-affirmation buffers negative consequence of the feeling of financial deprivation on delay discounting. Furthermore, in contrast to the participants in the financially deprived condition, self-affirmation did not influence participants’ delay discounting in the financially non-deprived condition. This is consistent with the logic of self-affirmation theory and a wealth of findings in this domain (see Cohen and Sherman, 2014) showing that affirmation interventions are most effective for people under psychological threat (e.g., the financially deprived).

#### Testing the Mediating Role of Sense of Personal Control

**FIGURE 3:**
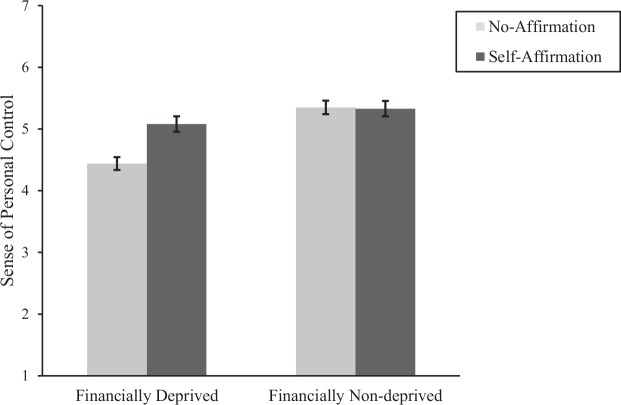
The sense of personal control as a function of experimental conditions in Study 2. Error bars denote ±1 SEM.

Results of a 2 (financial status: deprived vs. non-deprived) × 2 (affirmation: self-affirmation vs. no-affirmation) between-subjects ANOVA on participants’ sense of personal control revealed a main effect of financial status, *F*(1,428) = 28.75, *p* < 0.001, η^2^*_*p*_* = 0.06, a main effect of affirmation, *F*(1,428) = 8.32, *p* = 0.004, η^2^*_*p*_* = 0.02, and the critical two-way interaction between financial status and affirmation, *F*(1,428) = 9.25, *p* = 0.002, η^2^*_*p*_* = 0.02 (see Figure 3).

Analysis of the simple effects revealed that, in the deprived condition, participants who self-affirmed perceived themselves to have a higher sense of personal control (M = 5.08, SD = 1.17) than did those in the no-affirmation condition, M = 4.44, SD = 1.22; *F*(1,428) = 18.14, *p* < 0.001, *d* = 0.53, 95% CI _Mean–Difference_ [0.34, 0.93]. However, in the non-deprived condition, there was no significant difference in the perceived sense of control between those who affirmed (M = 5.33, SD = 1.03) and those who did not, M = 5.35, SD = 1.03; *F* < 1, *p* = 0.91, *d* = 0.02, 95% CI _Mean–Difference_ [−0.32, 0.29], suggesting that self-affirmation did not further boost a sense of control among participants who were not feeling financially deprived. Overall, these results are consistent with our reasoning that self-affirmation promotes a sense of personal control among the financially deprived (see Table 3 for summary statistics). We, therefore, proceeded to test whether the effect of self-affirmation on delay discounting of the financially deprived is statistically mediated through the sense of control.

**TABLE 3:** Summary statistics of the main outcome variables in Study 2, as a function of financial status and affirmation experimental conditions.

	**Deprived**		**Non-deprived**	
	**No-affirmation**	**Self-affirmation**	**No-affirmation**	**Self-affirmation**
AUC	0.32 (0.20)	0.45 (0.26)	0.45 (0.23)	0.44 (0.24)
Sense of personal control	4.44 (1.22)	5.08 (1.17)	5.35 (1.03)	5.33 (1.03)
Positive affect	2.84 (0.91)	2.99 (0.98)	3.02 (0.88)	3.24 (0.94)
Negative affect	1.79 (0.91)	1.66 (0.80)	1.4 (0.59)	1.53 (0.76)
Cell size (*n*)	116	107	103	106

*Each cell shows the mean and the standard deviation of the respective measure. Numbers in the parentheses represent standard deviations (SD).*

We used Hayes’s (2017) PROCESS macro (model 8) to test this proposition. A 10,000-resampled percentile bootstrap revealed a significant indirect effect of financial status × affirmation on delay discounting via sense of control, *index of moderated mediation* = −0.022, SE_bootstrap_ = 0.009, 95% CI [−0.043, −0.006]). As expected, for participants in the financially deprived condition, sense of control mediated the effect of self-affirmation (vs. no-affirmation) on delay discounting, *b* = 0.22, SE = 0.008, 95% CI [0.007, 0.040]. However, this was not the case for participants in the financially non-deprived condition, *b* = −0.001, SE = 0.005, 95% CI [−0.010, 0.010]. Overall, these findings support our second hypothesis (H2) that self-affirmation boosts a sense of personal control—a self-view that is capable and resourceful in overcoming challenges and constraints—among those who feel financially deprived, which in turn reduces their delay discounting tendency.

#### Testing the Mediating Role of Affective States

Results of a 2 (financial status: deprived vs. non-deprived) × 2 (affirmation: self-affirmation vs. no-affirmation) between-subjects ANOVA on participants’ *positive affect* revealed a main effect of financial status, *F*(1,428) = 5.92, *p* = 0.015, η^2^*_*p*_* = 0.01, such that on average participants who were in the deprived condition (M = 2.91, SD = 0.95) felt less positive than their non-deprived counterparts did (M = 3.13, SD = 0.92). Results also revealed a main effect of affirmation, *F*(1,428) = 4.02, *p* = 0.046, η^2^*_*p*_* = 0.01, such that on average participants in the self-affirmation condition (M = 3.11, SD = 0.97) felt more positive than those in the no-affirmation condition did (M = 2.93, SD = 0.90). However, the interaction between financial status and affirmation was not significant, *F* < 1, *p* = 0.69, suggesting that these two factors did not have a unique, joint effect on positive affect (see Table 3 for summary statistics). Consequently, it is unlikely that positive affect mediates the interaction between financial status and affirmation on delay discounting. To confirm this, a follow-up analysis revealed that when we controlled for participants’ positive affect, the interaction between financial status and affirmation on delay discounting remained significant, *F*(1,427) = 8.57, *p* = 0.004, η^2^*_*p*_* = 0.02. However, positive affect was not a significant predictor of delay discounting, *F* < 1, *p* = 0.73.

In a similar vein, results of a 2 (financial status: deprived vs. non-deprived) × 2 (affirmation: self-affirmation vs. no-affirmation) between-subjects ANOVA on participants’ *negative affect* revealed only a main effect of financial status, *F*(1,428) = 12.39, *p* < 0.001, η^2^*_*p*_* = 0.03, such that on average participants in the deprived condition (M = 1.73, SD = 0.86) felt more negative than their non-deprived counterparts did (M = 1.46, SD = 0.68). However, neither the main effect of affirmation condition, *F* < 1, *p* = 0.98, nor its interaction with financial status were significant, *F*(1,428) = 2.76, *p* = 0.097, η^2^*_*p*_* = 0.01, suggesting that self-affirmation did not influence participants’ negative affect in our sample (see Table 3 for summary statistics). It is, therefore, unlikely that negative affect mediates the interaction between financial status and affirmation on delay discounting. Confirming this, a follow-up analysis revealed that when we controlled for participants’ negative affect, the interaction between financial status and affirmation on delay discounting remained significant, *F*(1,427) = 8.40, *p* = 0.004, η^2^*_*p*_* = 0.02. However, negative affect was not a significant predictor of delay discounting, *F* < 1, *p* = 0.87.

### Discussion

The results of Study 2 conceptually replicate our findings in Study 1 and show that self-affirmation buffers the negative consequences of financial deprivation on delay discounting (H1). Furthermore, corroborating our H2, results of this study suggest that a boost in the sense of personal control, rather than change in affective states, provides a more parsimonious and plausible explanation on how self-affirmation reduces delay discounting of the financially deprived.

## General Discussion and Future Research Directions

Lack of financial resources increases delay discounting, a tendency that characterizes myopic behavior of the financially deprived across various decision domains, ranging from finances to education and health (Haushofer and Fehr, 2014; Farah and Hook, 2017; Pepper and Nettle, 2017). Bridging recent findings on the psychology of poverty and the self-affirmation theory, we proposed and provided evidence from two empirical studies that self-affirmation reduces delay discounting of the financially deprived. Specifically, across multiple operationalizations of financial deprivation (Study 1: income, Study 2: feeling of financial deprivation), we showed that the mitigating effect self-affirmation (manipulated) on delay discounting of the financially deprived was robust and remained significant even after controlling for participants’ demographic and socioeconomic characteristics (Studies 1 and 2). Moreover, consistent with our reasoning, we found that the effect of self-affirmation in reducing delay discounting of the financially deprived is driven by an increase in the sense of personal control (Study 2). Our findings also show that change in participants’ affective state—either positive or negative—did not explain the effect of self-affirmation on delay discounting of the financially deprived, thus ruling out an affect-based explanation for our findings (Studies 1 and 2).

Our research contributes to the psychology of poverty as well as the self-affirmation theory in several important ways. First, even though the relationship between financial deprivation and increased delay discounting is well-established in the poverty literature (Haushofer and Fehr, 2014; Pepper and Nettle, 2017), little is known about the psychological drivers of this effect and critically about the interventions that might mitigate this effect. The present research contributes to this literature by highlighting the self-threatening nature of lacking financial resources in explaining the delay discounting of the financially deprived and by identifying self-affirmation as a psychological remedy for this counterproductive tendency.

Second, we contribute to the self-affirmation theory by showing that the reparative effects of self-affirmation extend to the decision domains that are directly tied to the economic behavior of people under self-threat, a topic which has received scant attention in the psychology of self-defense. Specifically, whereas the past findings have highlighted cognitive (Hall et al., 2014), academic (Goyer et al., 2017), and health-related (Armitage et al., 2008) benefits of self-affirmation for the financially deprived, our research shows that self-affirmation can also enable the financially deprived to forego smaller-sooner payoffs and wait for larger-later payoffs (i.e., decreased delay discounting). Importantly, the ability to wait for larger-later payoffs is associated with a constellation of positive tendencies that can improve the poor’s well-being, such as accruing more savings and having less debt, and adopting healthier consumption habits (Mischel, 2014).

From a practical and policy perspective, it is important to emphasize that our research does not imply that psychological interventions, such as self-affirmation, can or should replace the benefits of interventions and programs entailing financial support in alleviating poverty. Rather, the culmination of our work is that affirmation interventions can *enable* the financially deprived to detach themselves, *at least momentarily*, from their threatening state and make decisions in agreement with their long-term interests. Consequently, our suggestion is to incorporate empirically verified psychological interventions, which often do not require hefty investments and are easy to implement, into more comprehensive programs aiming at alleviating poverty.

Our findings also provide new avenues for future research. Specifically, concerning the underlying process, although our findings are consistent with several research in the past showing that self-affirmation buffers negative consequences of psychological threats by cultivating a sense of control and agency (Reed and Aspinwall, 1998; Harris et al., 2007; Armitage et al., 2008; Morgan and Harris, 2015; Albalooshi et al., 2019), two important questions remain unanswered which future research may profitably explore. First, how does a sense of personal control enhance future-oriented decision-making among the financially deprived? Second, what are the other control-restoring mechanisms that may reduce delay discounting of the financially deprived?

Concerning the first question, we contend that increased sense of personal control may enhance executive functions of the financially deprived, which in turn improves future-oriented decision-making. Our speculation is based on findings that highlight a direct association between the sense of control and executive functions on the one hand, and between executive functions and self-regulation on the other hand. Specifically, lack of control has been found to impair executive functions (Smith et al., 2008), which in turn hampers self-regulatory abilities (Hofmann et al., 2012). Notably, impaired executive functions are associated with increased delay discounting (Hinson et al., 2003) and impulsivity (Bickel et al., 2012). In contrast, an increased sense of control has been found to improve executive functions (Albalooshi et al., 2019), which subsequently foster goal-directed behavior and planning (Diamond, 2013). Accordingly, increased sense of control and agency, resulting from self-affirmation, may foster future-oriented tendencies of the financially deprived by improving executive functions. This intriguing possibility also implies that the motivational and cognitive routes through which self-affirmation extends its reparative effects among the poor may be interrelated. Future research can fruitfully investigate these possibilities.

Future research can also investigate other factors that might enhance the sense of control of the financially deprived. Specifically, self-affirmation is inherently an *intrapersonal* intervention that boosts a resourceful self-view through affirming core personal values. Future research, however, can examine the role of control-restoring interventions, which are *interpersonal* in nature. For instance, research has found that people with strong social capital are shielded from environmental stressors (Ensel and Lin, 1991; Harber et al., 2008; Schnall et al., 2008). This is because social systems are sources of power and control (Portes, 1998; Adler and Kwon, 2002). Social capital, therefore, might mitigate the detrimental effects of financial deprivation on decision-making. This conjecture is consistent with recent research showing that trust in one’s community—the belief that one is surrounded by trustworthy people—offsets the negative effect of low income on myopic decisions (Jachimowicz et al., 2017).

The present work has limitations that future research can help address. First, future research may conceptually replicate and extend our work using different self-affirmation interventions. For example, when affirming their self-worth, people may focus on their independent (e.g., agency or analytical skills; SimanTov-Nachlieli et al., 2017) or interdependent (e.g., belonging or kindness; Shnabel et al., 2013) self-aspects and attributes. An interesting extension of our work may be to examine and compare the effectiveness of different types of self-affirmations in improving the future-oriented decision-making of the financially deprived. Furthermore, research may conceptually replicate our work using different manifestations of our dependent variable in decision domains that are indispensable for well-being of the financially deprived, such as the choice between healthy and unhealthy food options (Briers and Laporte, 2013) or between saving or spending resources (Steinhart and Jiang, 2019).

Finally, future research can also examine whether the mitigating effect of self-affirmation on delay discounting of the poor is enduring over time. Whereas our findings cannot speak to this point, research on the self-affirmation literature has provided evidence on the long-term benefits of affirmation interventions on various academic outcomes of low-income and other stigmatized groups (Sherman et al., 2013; Cohen and Sherman, 2014; Goyer et al., 2017). It is, therefore, promising for future research to extend our findings by examining the enduring effect of self-affirmation interventions in mitigating myopic tendencies of the financially deprived across various decision domains.

## Conclusion

In the present work, we argued that the self-threatening nature of lack of financial resources may be one driver of how financial deprivation increases delay discounting. Accordingly, we proposed and provided evidence that self-affirmation—an intervention known to counter the effect of psychological threats—buffers the detrimental effect of financial deprivation on delay discounting. By reminding people of psychological resources at their disposal, self-affirmation bolsters a sense of personal control among the financially deprived, which in turn enables them to wait for larger-later payoffs. Overall, our findings suggest that the relationship between financial deprivation and delay discounting is malleable and psychological interventions that buffer self-threats and bolster a sense of personal control can be successfully applied to reduce myopic tendencies of the poor.

## Ethics Statement

All participants gave their informed consent in accordance with the Declaration of Helsinki, before participating in the pretest or the main experiments and their data were treated anonymously. This research was conducted according to the guidelines provided by the Research Ethics Committee (REC) of the Faculty of Economics and Business, University of Groningen. Additionally, prior to data collection, the REC reviewed and approved this research and its experimental procedures.

## Author Contributions

All co-authors contributed significantly at different stages of this research. In particular, MM-J and SA developed the initial research idea and hypotheses and analyzed the data. All authors designed and preregistered the experiments on Open Science Framework. MM-J conducted the experiments, wrote the manuscript, and prepared the Supplementary Material. SA and IS revised and provided the critical feedback on the manuscript and the Supplementary Material.

## Conflict of Interest Statement

The authors declare that the research was conducted in the absence of any commercial or financial relationships that could be construed as a potential conflict of interest.

## Footnotes

1 https://osf.io/8kpur (Study 1) and https://osf.io/kyfw6/ (Study 2).

2 In handling the open-bound income categories, some researchers consider the lower limit of the highest category (e.g., $190,000) as the corresponding income amount. We find similar results using that approach.

3 Descriptive statistics of our samples in Studies 1 and 2 with respect to income and other demographic variables have been provided in the Supplementary Material. Furthermore, we did not find any significant difference in age, gender, income, ethnicity, education level, employment status, and household size across experimental conditions, confirming a successful random assignment in both studies.

4 Income values, based on the midpoint of the income bracket participants chose (M = $47,436, SD = 35,666), ranged from $5,000 to $195,000 [IQR: 25,000–65,000]. Using log-transformed income values in our analyses does not change the pattern or the significance of our findings.

5 Prior to this experiment, we conducted an independent pretest to validate and confirm the effectiveness of our financial status manipulation in inducing the feeling of financial deprivation. To accurately assess the differential effects of our focal experimental conditions (i.e., financially deprived, and non-deprived) on participants’ subjective perception of financial status, we also included a *control* condition in this pretest, in which financial thoughts or concerns were not provoked among participants. Results of the pretest showed that the observed effect on subjective perception of financial status was driven only by responses in the financially deprived condition and that our manipulation worked successfully and as intended to induce feeling of financial deprivation among participants. See Supplementary Material for details of this pretest.

6 The crtical interaction between financial status (manipulated) and self-affirmation (manipulated) was robust and remained significant even after controlling participants’ socieconomic and demographic characterisctics (e.g., age, gender, income, ethnicity, level of education, employment status, and household size). Details of our robutness tests can be found in the Supplementary Material.
