# Supplementary material for: Self-Affirmation Reduces Delay Discounting of the Financially Deprived
Source: Front Psychol. 2019 Jul 30;10:1729. doi: 10.3389/fpsyg.2019.01729 (PMC6682614; doi:10.3389/fpsyg.2019.01729)
Supplement: Supplementary file 1 [file Data_Sheet_1.pdf]

Supplementary Material

"Self-Affirmation Reduces Delay Discounting of the Financially Deprived"

Mehrad Moeini-Jazani[1*], Sumaya Albalooshi[1], Ingvild Müller Seljeseth[2,3]

[1]University of Groningen, Groningen, The Netherlands

[2]Kristiania University College, Oslo, Norway

[3]BI Norwegian Business School, Oslo, Norway

* *Correspondence:* Mehrad Moeini-Jazani, Faculty of Economics & Business, University of Groningen, Nettelbosje 2, 9747 AE Groningen, Netherlands. E-mail: m.moeini.jazani@rug.nl.

Note: This document has two main sections. In section 1, we have disclosed all materials and measures used in our experiments. Information is presented according to the order by which the studies and instructions have been mentioned in the manuscript. In section 2, we have provided additional analyses of our data, including the results of the pretest for our financial deprivation manipulation in Study 2, as well as the results of robustness tests for our findings in Study 2.

SECTION 1: EXPERIMENTAL PROCEDURES AND MATERIALS

Study 1. Income, Self-affirmation, and Delay Discounting

A. Affirmation Manipulation

We followed the standard value-affirmation procedure (Cohen et al., 2006; McQueen & Klein, 2006; Sherman et al., 2009). All participants were first asked to rank the following list of 12 values in order of their importance to them. Specifically, they completed the following:

"Below is a list of characteristics and values, some of which may be important to you, some of which may be unimportant. Please rank these values and qualities in order of their importance to you, from 1 to 12 (1 = most important item, 12 = least important item)"

______ Artistic skills/aesthetic appreciation

______ Sense of humor

______ Relations with friends/family

______ Spontaneity/living life in the moment

______ Social skills

______ Athletics

______ Musical ability/appreciation

______ Physical attractiveness

______ Creativity

______ Analytical skills

______ Romantic values

______ Contentment and gratefulness

Participants in the *self-affirmation* condition were then asked to think about why their top-ranked value was important to them. Specifically, they received the following instructions:

> “Now that you ranked your values, please write about why your most valued characteristic, the item you ranked "1", is personally important to you and describe a time when it had been particularly important to you.”

Participants in the *no-affirmation* condition wrote about why the value they ranked 12th might be important to an average university student. Specifically, they received the following instructions:

> “Now that you ranked the values, please briefly describe a situation where your least valued characteristic (the item you ranked "12") might be important to an average university student.”

B. Positive and Negative Affect Schedule (PANAS)

To capture participants’ positive and negative affective states, we used the PANAS scale (Watson et al., 1988), which consists of 20 items (10 related to the positive and 10 related to the negative affect). Participants completed the PNASA using 5-point scales (1= not at all, 5 = extremely).

C. Delay Discounting

We used three questions to elicit participants tendency to discount delayed payoffs. Specifically, participants indicated the amount of money (in U.S. dollars) they would require in 3, 9, and 18 months in the future, to make them indifferent to receiving $65 now. These questions were presented individually, in random order. In the following, we have provided one of these questions as an illustration.

> Imagine that you can receive **$65 Now**. How much money do you require instead to wait for **nine months**? Please indicate the amount in the box provided.
>
> **$65 now = $____ in 9 months.**

D. Income and Demographic Variables

In the last part of the survey, we measured demographic variables. In particular, we measured participants' income using 20 income brackets with $10,000 increments, ranging from 1 (under $10,000) to 20 (190,000 or more). In addition to income, we measured other demographic characteristics that may covary with income. These included: *age*, *gender*, *ethnicity* (1 = European-American, 2 = African-American, 3 = Hispanic or Latin-American, 4 = Asian-American, 5 = Native-American, 6 = Pacific Islander, 7 = other), *level of education* (1 = less than high school, 2 = high school diploma or GED, 3 = associate or vocational degree , 4 = college or university degree, 5 = master's degree, 6 = Doctoral student or holder of a doctoral degree, 7 = other), and *employment status* (1 = full-time, 2 = part-time, 3 = self-employed, 4 = unemployed but looking for a job, 5 = retired, 6 = housewife or househusband, 7 = unable to work/other) and *household size*. Finally, participants answered the following attention check question, which was included to assess participants' attentiveness to instructions (Oppenheimer et al., 2009).

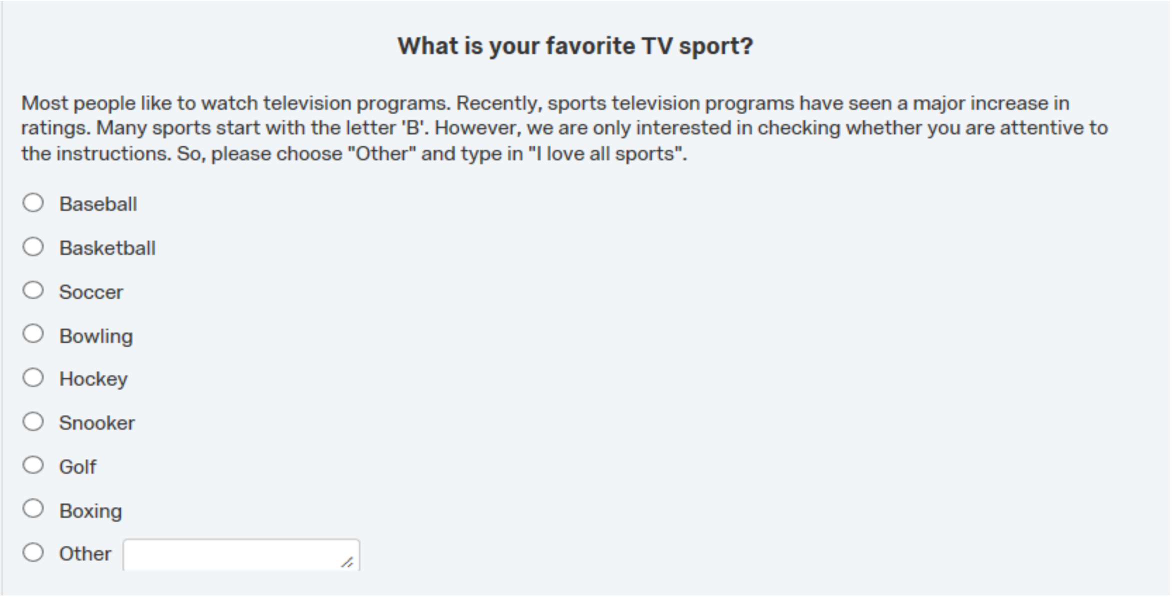

What is your favorite TV sport?

Most people like to watch television programs. Recently, sports television programs have seen a major increase in ratings. Many sports start with the letter 'B'. However, we are only interested in checking whether you are attentive to the instructions. So, please choose "Other" and type in "I love all sports".

- Baseball
- Basketball
- Soccer
- Bowling
- Hockey
- Snooker
- Golf
- Boxing
- Other

Study 2. Feeling of Financial Deprivation, Self-affirmation, and Delay Discounting

A. Financial Deprivation Manipulation

After participants indicated their age, gender, and household size, they were randomly assigned to one of the two financial status conditions: *deprived* or *non-deprived*. In both conditions, participants were first asked to indicate their monthly income level using a response scale provided below.

In the *financially non-deprived* condition, participants used the following response scale:

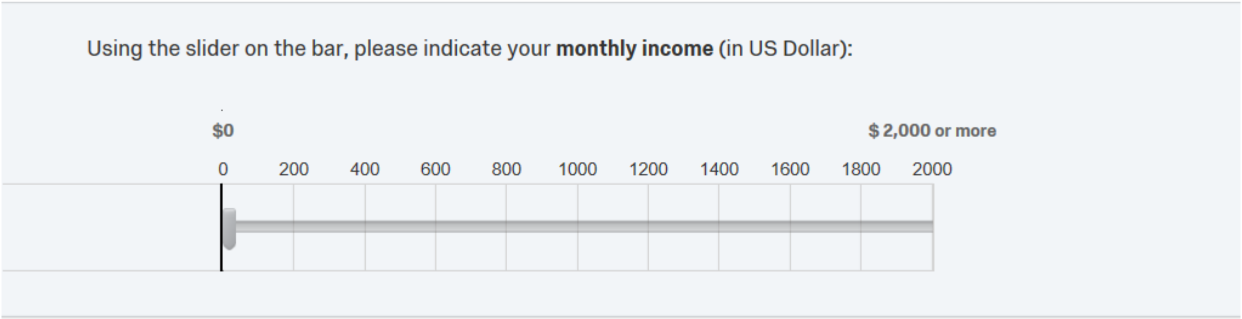

In the *financially deprived* condition, participants used the following response scale:

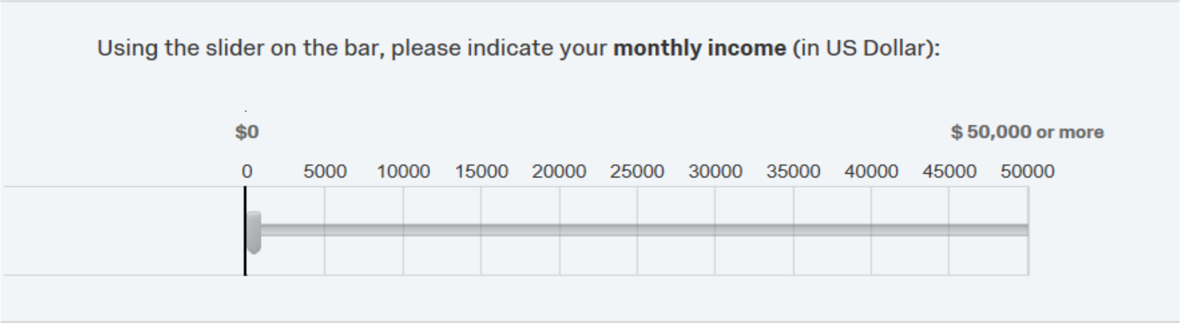

Next, all participants were informed that an algorithm would calculate their relative financial status, by comparing their income with people who match their profile in a large and representative national sample of individuals' income data. Once participants clicked "continue," they viewed an animated loading bar with a text "please wait a few seconds while the system calculates your relative financial status and well-being…", created to make the impression that the computer is accessing the database and processing information.

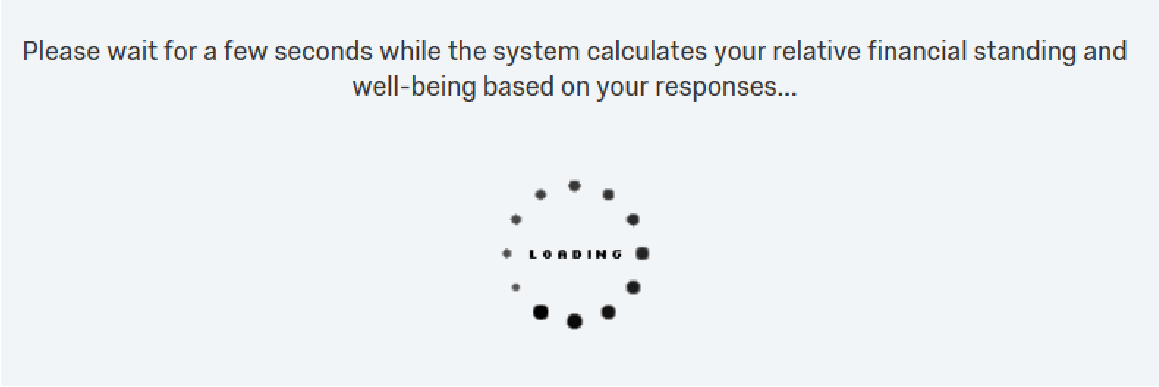

Subsequently, participants received bogus feedback about their financial status, corresponding to their assigned experimental conditions. Particularly, participants in the *financially non-deprived* condition received the following feedback:

> "Our online calculator compared your information and income with a large, representative database of individuals who have a similar profile as you do. Based on the information you provided, our calculator identified you as an individual who is financially adequate, relative to others; that is, someone who, relatively, has adequate and sufficient financial resources (i.e., money). We would like you to take a few minutes to reflect and write on how it feels to be in a relatively adequate financial position and to know that, on average, you have sufficient money to use at your will or when required in daily life, relative to those who are financially more constrained. Consider carefully and vividly how your life is with a relatively adequate amount of money and what the consequences of having sufficient money to live a stable life are."

In contrast, participants in the *financially deprived* condition received the following feedback:

> "Our online calculator compared your information and income with a large, representative database of individuals who have a similar profile as you do. Based on the information you provided, our calculator identified you as an individual who is financially constrained, relative to others; that is, someone who may experience financial difficulties and, relatively, lack adequate financial resources (i.e., money). We would like you to take a few minutes to reflect and write on how it feels to be in a relatively inadequate financial position and to know that, on average, you might not have sufficient money to use at your will or when required in daily life, relative to those who are financially less constrained. Consider carefully and vividly how your life is with a relatively inadequate amount of money and what the consequences of not having sufficient money to live a stable life are."

B. Affirmation Manipulation

We used the same procedure outlined in Study 1.

C. Measuring Potential Mediating Variables

We measured participants' feeling of control using the *personal control* scale (Lachman & Weaver, 1998). This scale consists of 12 statements, and participants rated those

statements using 7-point scales (1= strongly disagree, 7= strongly agree). The order by which these items were presented was randomized in our experiment. To capture participants' positive and negative affective states, we used the PANAS scale (Watson et al., 1988), which consists of 20 items (10 related to the positive and 10 related to the negative affect). Participants completed the PNASA using 5-point scales (1= not at all, 5 = extremely).

D. Delay Discounting

We used the same procedure outlined in Study 1 to measure participants' delay discounting.

E. Measuring Demographic Variables

We measured participants' income and demographic characteristics (e.g., ethnicity, level of education, and employment status) using the same scales used in Study 1. As highlighted in our preregistered plan, we measured these variables to test the robustness of our main findings. Finally, participants answered the following attention check question (Oppenheimer et al., 2009).

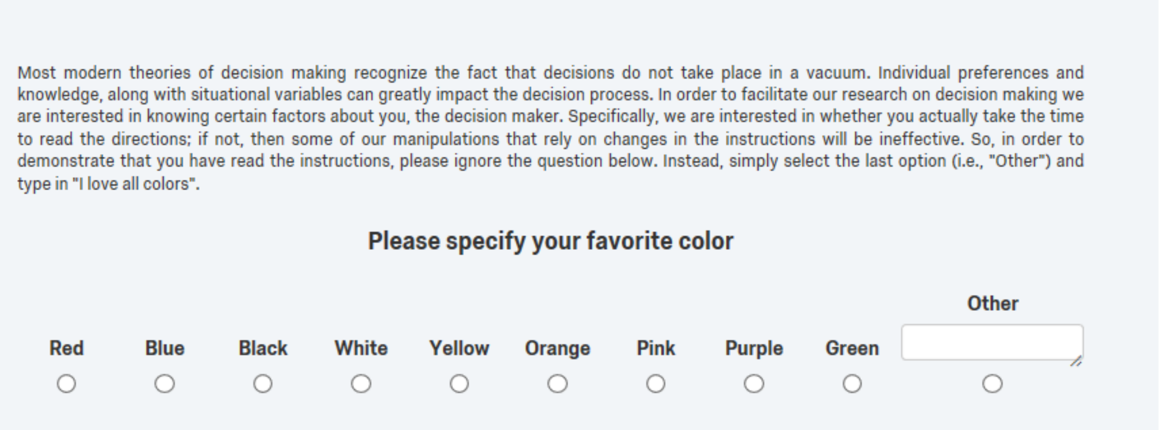
Most modern theories of decision making recognize the fact that decisions do not take place in a vacuum. Individual preferences and knowledge, along with situational variables can greatly impact the decision process. In order to facilitate our research on decision making we are interested in knowing certain factors about you, the decision maker. Specifically, we are interested in whether you actually take the time to read the directions; if not, then some of our manipulations that rely on changes in the instructions will be ineffective. So, in order to demonstrate that you have read the instructions, please ignore the question below. Instead, simply select the last option (i.e., "Other") and type in "I love all colors".

Please specify your favorite color

Red Blue Black White Yellow Orange Pink Purple Green Other

SECTION 2: DEMOGRAPHIC CHARACTERISTICS OF THE SAMPLES, PRETEST RESULTS AND ROBUSTNESS CHECKS FOR STUDY 2

In this section, we provide descriptive characteristics of our participants' demographics across both studies. We then present the results of the pretest which we conducted to validate the effectiveness of our financial deprivation manipulation in Study 2. Furthermore, we present the results of robustness tests concerning our findings in Study 2.

Study 1. Demographics

Participants annual income, based on the midpoint of the bracket they chose (M = $47,436, SD = 35,666), ranged from $5,000 to $195,000 [*IQR*: 25,000 to 65,000], and was non-normally distributed with skewness of 1.14 (SE = 0.105). Overall, 417 (76.4%) participants in our sample were European-American, 45 (8.2%) were African-American, 31 (5.7%) were Hispanic, 37 (6.8%) were Asian-American, 8 (1.5%) were Native-American, and 8 (1.5%) had other ethnic backgrounds. Furthermore, 2 (0.4%) participants had educations below high school level, 129 (23.6%) had a high school diploma or GED, 111 (20.3%) had an associate or vocational degree, 227 (41.6%) had a college or university degree, 66 (12.1%) had a master's degree, and 11 (2%) had higher degrees. With respect to the employment status, 301 (55.1%) participants were full-time employed, 66 (12.1%) were part-time employed, 67 (12.3%) were self-employed, 33 (6%) were looking for a job, 23 (4.2%) were retired, 34 (6.2%) were homemakers, and finally 22 (4%) were unable to work.

Study 2. Demographics

Participants annual income, based on the midpoint of the bracket they chose (M = $ 49, 395, SD = 34,395), ranged from $7,500 to $185,000 [*IQR*: 30,000 to 60,000], and was non-

normally distributed with skewness of 1.31 (*SE* = 0.118). Overall, 334 (77.3%) participants in our sample were European-American, 37 (8.6%) were African-American, 25 (5.8%) were Hispanic, 29 (6.7%) were Asian-American, 1 (0.2%) was Native-American, and 6 (1.4%) had other ethnic backgrounds. Furthermore, 115 (26.6%) participants had a high school diploma or GED, 7 (1.6%) had an associate or vocational degree, 239 (55.3%) had a college or university degree, 61 (14.1%) had master's degree, and 10 (2.3%) had higher degrees. With respect to the employment status, 255 (58.3%) participants were full-time employed, 47 (11.6%) were part-time employed, 46 (10.6%) were self-employed, 25 (5.8%) were looking for a job, 13 (3%) were retired, 34 (7.9%) were homemakers, and finally 12 (2.8%) were unable to work.

Validation of the Financial Deprivation Manipulation: Pretest

Prior to the actual experiment, we conducted an independent pretest to validate and confirm the effectiveness of our financial status manipulation in inducing the feeling of financial deprivation. Using TurkPrime, we recruited 296 American participants from MTurk who were randomly assigned to one of the *three* experimental conditions: *financially deprived, financially non-deprived,* and the *control* condition. Participants in the *financially deprived and non-deprived* conditions went through the same procedure outlined in the manuscript before responding to a 4-item questionnaire which assessed their subjective perception of financial status[1]. In contrast, participants in the *control* condition were only asked to write about two facts that they knew of before they completed the 4-item questionnaire about their financial status.

---

[1] These questions were the following (1) "As you completed this study, to what extent did you feel financially constrained?" (1= not at all, 7 = very much so); (2) "How would you rate your financial position in comparison to your peers' financial position?" (1= much worse, 7= much better); (3) "How would you rate your ability to spend money freely compared to your peers' ability to spend money freely?" (1 = much worse, 7 = much better); and (4) "Overall, how would you rate your financial satisfaction?" (1= not at all satisfied, 7 = very satisfied). After reverse coding the first item, all items (α = .90) were averaged to form a single manipulation check measure, scored such that higher ratings indicated greater perceived financial status.

Therefore, financial thoughts or concerns were not provoked among these participants. This allowed us to accurately assess the differential effects of our focal experimental conditions (i.e., financially deprived, and financially non-deprived) on participants' subjective perception of financial status, by comparing each condition with the control condition. After completing the 4-item questionnaire, all participants answered demographic questions (e.g., ethnicity, level of education, employment status, and annual income level) followed by an attention check question (Oppenheimer et al., 2009), were debriefed, and paid.

We excluded 14 participants who did not complete the pretest survey ($n = 4$) or failed the attention check question ($n = 10$). The pretest analysis therefore was conducted on the remaining 282 participants ($M_{\text{age}} = 34.73$, SD = 11.83; 172 females). A one-way ANOVA revealed a significant effect of condition on subjective perception of financial status, $F(2, 279) = 23.84$, $p < 0.001$, $\eta^2_p = 0.15$. Planned contrasts revealed that participants in the financially-deprived condition ($M = 3.12$, $SD = 1.46$) perceived to have worse financial status than did those in the financially non-deprived, ($M = 4.28$, $SD = 1.08$; $F(1, 279) = 39.54$, $p < 0.001$, $d = 0.90$, 95% CI $_{\text{Mean-Difference}}$ [-1.53, -0.80]), and the control conditions, $M = 4.15$, $SD = 1.23$; $F(1, 279) = 31.31$, $p < 0.001$, $d = 0.76$, 95% CI $_{\text{Mean-Difference}}$ [-1.39, -0.67]. There was no significant difference in subjective perception of financial status between participants in the financially non-deprived and the control conditions ($F < 1$, $p = 0.48$, 95% CI $_{\text{Mean-Difference}}$ [-0.23, 0.49]), suggesting that participants in the non-deprived condition did not feel financially superior to those in the control condition. Together, these results show that the observed effect was driven only by participants' responses in the financially deprived condition and that our manipulation worked successfully and as intended to induce feeling of financial deprivation[2].

[2] Controlling for participants' demographic characteristics (e.g., age, gender, household size, ethnicity, level of education, employment status) and annual income did not change the pattern or significance of our pretest results,

Robustness Tests for Study 2

Using a series of regression analyses (see Table S.1), we tested whether the focal interaction between our main factors, financial status, and affirmation (Model 1), on AUC remained significant after controlling for participants' income (Model 2) and demographic characteristics (Model 3) that may covary with income (e.g., ethnicity, level of education, employment status, and household size). As these results in Table S1 show, the critical interaction between financial status and affirmation on AUC remained significant even after controlling participants' socioeconomic and demographic characteristics. These results, therefore, corroborate the robustness of our main findings in Study 2.

further confirming the effectiveness of our procedure in manipulating participants' perception of their financial status.

Table S.1.

Robustness Tests for Study 2

	Model 1			Model 2			Model 3		
Variable	*b*	SE_b	*t*	*b*	SE_b	*t*	*b*	SE_b	*t*
Intercept	0.321	0.022	14.69***	0.352	0.027	13.12***	0.321	0.042	7.70***
Financial Status	0.128	0.032	4.03***	0.106	0.032	3.31***	0.108	0.032	3.37***
Affirmation	0.125	0.032	3.97***	0.114	0.031	3.65***	0.115	0.031	3.68***
Financial Status × Affirmation	-0.132	0.045	-2.92**	-0.111	0.045	-2.47*	-0.116	0.045	-2.57*
Age				0.003	0.001	2.91**	0.003	0.001	3.00**
Gender				-0.035	0.023	-1.53	-0.034	0.023	-1.47
Income Level				0.008	0.003	2.39*	0.006	0.003	1.66†
Ethnicity							0.029	0.027	1.08
Education Level							0.016	0.011	1.54
Employment Status							0.009	0.029	0.32
Household Size							0.011	0.008	1.34
Adjusted R^2	.046			.076			.081		
R^2 change				.037***			.013*n.s.*		

Note: The area under the discounting curve (AUC) serves as the dependent variable. Regression coefficients are unstandardized. For model 1, *n* = 432. For model 2 and 3, *n* = 423 because we excluded participants who had preferred not to indicate their income levels (*n* = 6) or they had not classified their gender as male or female (*n* = 3). Financial status was coded 1 = non-deprived, and 0 = deprived. Affirmation was coded 1 = self-affirmation, and 0 = no-affirmation. Gender was coded 1 = female and 0 = male. Ethnicity was coded 1 = European-American, and 0 = others. Employment status was coded 1 = full-time, part-time, or self-employed, and 0 = unemployed. Income was divided by 10,000 and then centered at its grand mean Age, Education Level, and Household Size were centered at their grand means. *t* statistics are rounded to two digits after the decimal point. *n.s.* denotes not significant.
†$p < .10$, * $p < .05$, ** $p < .01$, *** $p < .001$.

References

Cohen, G. L., Garcia, J., Apfel, N., & Master, A. (2006). Reducing the racial achievement gap: A social-psychological intervention. *Science*, *313*(5791), 1307–1310. https://doi.org/10.1126/science.1128317

Lachman, M. E., & Weaver, S. L. (1998). The sense of control as a Moderator of Social Class Differences in Health and Well-Being. *Journal of Personality and Social Psychology*, *74*(3), 763–773.

McQueen, A., & Klein, W. M. P. (2006). Experimental manipulations of self-affirmation: A systematic review. *Self and Identity*, *5*(4), 289–354. https://doi.org/10.1080/15298860600805325

Oppenheimer, D. M., Meyvis, T., & Davidenko, N. (2009). Instructional manipulation checks: Detecting satisficing to increase statistical power. *Journal of Experimental Social Psychology*, *45*(4), 867–872. https://doi.org/10.1016/j.jesp.2009.03.009

Sherman, D. K., Bunyan, D. P., Creswell, J. D., & Jaremka, L. M. (2009). Psychological Vulnerability and Stress: The Effects of Self-Affirmation on Sympathetic Nervous System Responses to Naturalistic Stressors. *Health Psychology*, *28*(5), 554–562. https://doi.org/10.1037/a0014663

Watson, D., Clark, L. A., & Tellegen, A. (1988). Development and validation of brief measures of brief measures of positive and negative affect: the PANAS scales. *Journal of Personality and Social Psychology*, *54*(6), 1063–1070.
